# Supplementary material for: Intracerebral Injection of Extracellular Vesicles from Mesenchymal Stem Cells Exerts Reduced Aβ Plaque Burden in Early Stages of a Preclinical Model of Alzheimer’s Disease
Source: Cells. 2019 Sep 10;8(9):1059. doi: 10.3390/cells8091059 (PMC6770482; doi:10.3390/cells8091059)
Supplement: Supplementary file 1 [file cells-08-01059-s001.pdf]

Supplementary figure 1

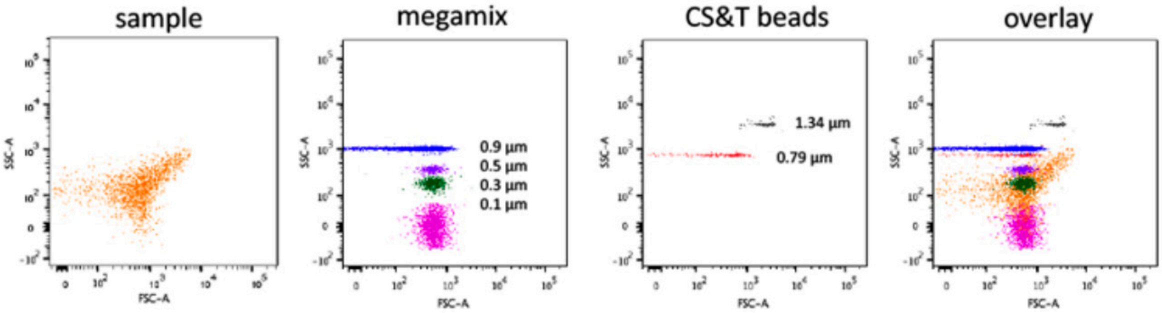

Sample panel: The EV population marked in flow cytometry by CD9 and CD49 antibodies is shown according to its physical parameters SSC-A and FCS-A (orange). Megamix and CS&T beads panels show the distribution of megamix (Biocytex) and CS&T beads using the same voltage as for the physical parameters shown in the sample panel. Overlay panel shows the merge of the three previous panels indicating that EVs events measured by flow cytometry are mainly located above 0.1 µm and below 1.34 µm.
